# Machine learning and AI for cancer research and care: a review of applications, limitations, and future directions

**DOI:** 10.1186/s43046-026-00399-y

**Published:** 2026-08-14

**Authors:** Kanishk Yadav, Taneesha Gupta

**Affiliations:** 1 Division of Pediatric Cardiology, Department of Pediatrics, Duke University Health System, Durham, North Carolina USA; 2 Baptist Memorial Hospital, Tennessee, Memphis, USA

**Keywords:** Machine learning, Cancer research, Oncology, Genomics, Artificial intelligence, Deep learning, Precision medicine

## Abstract

Machine learning (ML) is transforming cancer research and care by enabling analysis of complex, high-dimensional datasets spanning genomics, transcriptomics, proteomics, imaging, and clinical records. By improving risk stratification, accelerating detection and diagnosis, and supporting treatment selection, ML has the potential to enhance survival outcomes while increasing efficiency across oncology workflows. This review synthesizes key developments in ML for oncology, covering foundational algorithms alongside emerging approaches. We highlight major application areas including early cancer detection, tumor classification, molecular subtyping, biomarker discovery, prognosis estimation, multi-omics integration, computational pathology, pharmacogenomics, and clinical decision support. We also summarize commonly used datasets, discuss the importance of interpretability for clinical trust, and outline barriers to translation such as data heterogeneity, bias, and regulatory constraints. Finally, we describe future directions, including federated learning, graph neural networks, longitudinal modeling, and integration of real-world and wearable data to support precision oncology. This review is intended for cancer researchers, clinicians, and data scientists seeking a practical overview of ML methods, opportunities, and translational considerations in oncology.

## Introduction

Cancer is one of the leading causes of death worldwide, accounting for nearly 10 million deaths in 2020 [1]. Its biological heterogeneity across tumor types, patients, and even within individual tumors complicates diagnosis, treatment, and prognosis. Advances in next-generation sequencing (NGS), high-throughput proteomics, digital pathology, and imaging have resulted in unprecedented volumes of complex data [2]. The need to extract clinically meaningful insights from these large, diverse datasets has positioned machine learning (ML) as a critical tool in modern oncology. 

ML, a subset of artificial intelligence, encompasses algorithms capable of learning patterns from data to make predictions or classifications without explicit rule-based programming [3]. Unlike traditional statistical methods, which often rely on predefined hypotheses, ML can capture nonlinear and high-dimensional relationships inherent in biomedical data. This capacity makes it especially suited to address challenges in cancer research, where the integration of multi-modal datasets is essential for comprehensive understanding.

In recent years, machine learning (ML) has enabled major advances across oncology, including:

- Automated tumor detection in radiological and histopathological images, with performance comparable to or exceeding expert clinicians [4, 5].
- Multi-omics integration (genomic, transcriptomic, epigenomic, and proteomic data) to uncover novel cancer subtypes and refine molecular stratification [6, 7].
- Drug response prediction and therapeutic target identification using pharmacogenomic datasets [8, 9].
- Clinical decision support to assist risk stratification and personalize treatment strategies [10].

Despite these successes, integrating ML into clinical workflows faces obstacles such as data quality, model interpretability, algorithmic bias, and regulatory constraints [11]. Unlike prior reviews that focus on a single modality (e.g., imaging or genomics) or emphasize algorithms in isolation, this review organizes ML advances across thematic clinical and translational domains, spanning multi-omics, computational pathology, pharmacogenomics, and decision support, while explicitly synthesizing barriers to deployment and outlining practical future research priorities.

Throughout the review, we use a pragmatic translational lens to separate retrospective proof-of-concept studies from externally validated, prospectively evaluated, and clinically interventional AI approaches, because these stages differ substantially in evidentiary strength and deployment readiness.

### Scope, conceptual framework, and study selection

This article is a narrative review informed by a targeted literature search rather than a systematic review. The goal was to synthesize clinically and translationally relevant developments in machine learning across the cancer-care continuum, with emphasis on studies that illustrate how AI methods move from data resources and model development toward diagnostic, prognostic, therapeutic, and workflow applications in oncology.

To inform this review, we searched PubMed, Scopus, and Web of Science, supplemented by Google Scholar and citation chaining, for English-language publications from January 1, 2015 through March 31, 2026 using combinations of terms related to cancer/oncology, machine learning/artificial intelligence/deep learning, screening, pathology, multi-omics, prognosis, drug response, clinical decision support, external validation, prospective evaluation, and randomized trial. We prioritized peer-reviewed primary studies, major benchmark resources, influential methodological papers, and reporting or evaluation frameworks relevant to clinical AI.

Inclusion criteria favored studies with clear oncologic relevance, meaningful validation design, and potential translational importance. Exclusion criteria included non-oncology applications, purely technical papers without clinical framing, conference abstracts lacking sufficient methodological detail, editorials, and studies with minimal description of the underlying cohort or validation strategy. Because the aim was integrative synthesis rather than exhaustive evidence mapping, we did not perform formal PRISMA-style screening, risk-of-bias scoring, or meta-analysis. Instead, we explicitly distinguish studies by clinical maturity, including retrospective internal validation, retrospective external or multicenter validation, prospective observational evaluation, and interventional or randomized clinical testing.

## Machine learning fundamentals for cancer research

### Categories of machine learning

ML approaches used in oncology can be broadly grouped into three categories: supervised learning, unsupervised learning, and reinforcement learning. Each is suited to different research questions and data structures, and they are often combined within a single pipeline to improve predictive performance and clinical relevance.

#### Supervised learning

Supervised learning models are trained on labeled datasets with known outcomes (e.g., tumor class, survival time, therapy response) and remain central to cancer classification, prognosis, and treatment prediction. Recent oncology work increasingly combines traditional supervised approaches for tabular clinical and omics features (regularized regression and tree-based ensembles) with advanced deep learning that can leverage transfer learning and multimodal fusion. For example, multimodal breast cancer survival models using TCGA BRCA integrate clinical variables with multiple molecular layers and digital histopathology, showing that late-fusion deep learning can improve prognostic performance and enable more robust risk stratification than single-modality models [12]. Similarly, multimodal survival frameworks such as SurvPGC incorporate pathology images, genomics, and clinically derived embeddings using cross-attention, demonstrating how supervised objectives can be strengthened by richer representations of low-dimensional clinical information [13].

A major shift in supervised oncology modeling is the adoption of foundation and pretraining-based strategies to reduce reliance on large labeled cohorts and improve generalizability. In computational pathology, CHIEF and related pathology foundation models learn transferable slide representations that can be adapted to downstream diagnostic and prognostic tasks, including survival prediction [14]. Self-supervised histopathology pretraining at scale (e.g., BEPH trained on millions of unlabeled images) further supports efficient adaptation to cancer classification and survival endpoints across tumor types [15]. Analogous trends are emerging in radiology, where cancer imaging foundation models trained with self-supervision improve downstream biomarker and endpoint modeling, especially when labeled data are limited [16]. In therapy modeling, supervised drug response prediction is also moving beyond earlier single-omics regressors toward multimodal deep learning that integrates multi-omics features with drug structure representations; for instance, PASO combines pathway-informed multi-omics features with SMILES-based drug encodings using transformers and attention for drug sensitivity prediction and clinical validation [17]. Together, these developments position supervised learning in oncology as an increasingly transfer-ready, multimodal, and clinically oriented paradigm [18].

#### Unsupervised learning

Unsupervised learning discovers structure in unlabeled datasets, making it valuable for exploratory analysis when tumor heterogeneity and incomplete labels limit predefined classification. In oncology, it is widely used for molecular subtype discovery, patient stratification, and representation learning for multi-omics integration. Recent work has moved beyond classical clustering and PCA toward deep clustering and graph-based methods that can capture nonlinear, cross-omics structure. For example, self-supervised multi-omics clustering models that combine graph convolutional networks with autoencoder representations can identify clinically meaningful subtypes with survival differences across cancers [19].

Common unsupervised tools still include clustering (K-means, hierarchical clustering) and dimensionality reduction for visualization, but many modern pipelines use deep representation learning before clustering. Contractive and other autoencoder variants can learn robust latent features from each omics layer, then cluster patients in the shared embedding to improve stability and subtype separation [20]. Contrastive and graph-based clustering is also increasingly used for multi-omics cancer subtyping, for example a multi-view, multi-level contrastive graph convolutional network that performs fusion-free representation learning and clustering across many TCGA multi-omics datasets [21]. At the single-cell level, foundation models trained with large-scale self-supervision (e.g., scGPT and CellFM) provide transferable embeddings that support unsupervised batch integration and discovery of cell states, offering a scalable route to characterize tumor microenvironment heterogeneity and generate hypotheses for downstream supervised modeling [22, 23].

#### Reinforcement learning

Reinforcement learning (RL) trains an agent to make sequential decisions by interacting with an environment and optimizing long-term reward, aligning well with time-dependent cancer care where treatment is adjusted across visits. In radiotherapy, deep RL has been applied to personalize fractionation schedules using simulated tumor-response environments, aiming to reduce toxicity while maintaining local control; recent examples include RL-driven de-escalation fractionation optimization for head and neck cancers and related RL frameworks for fractionation scheduling [24, 25]. RL is also being explored for therapy scheduling and dosing, including deep RL controllers for closed-loop chemotherapy dose adjustment in simulated patients [26], model-informed RL approaches to optimize combination treatment schedules in glioblastoma evolution [27], and RL-based personalization of intermittent androgen deprivation therapy strategies in prostate cancer [28].

While still less mature than supervised and unsupervised learning in clinical oncology, RL is a promising direction for adaptive treatment policies, but its translation depends on reliable simulators, careful off-policy evaluation, and safety-focused clinical validation.

### The machine learning workflow in cancer research

**Fig. 1:**
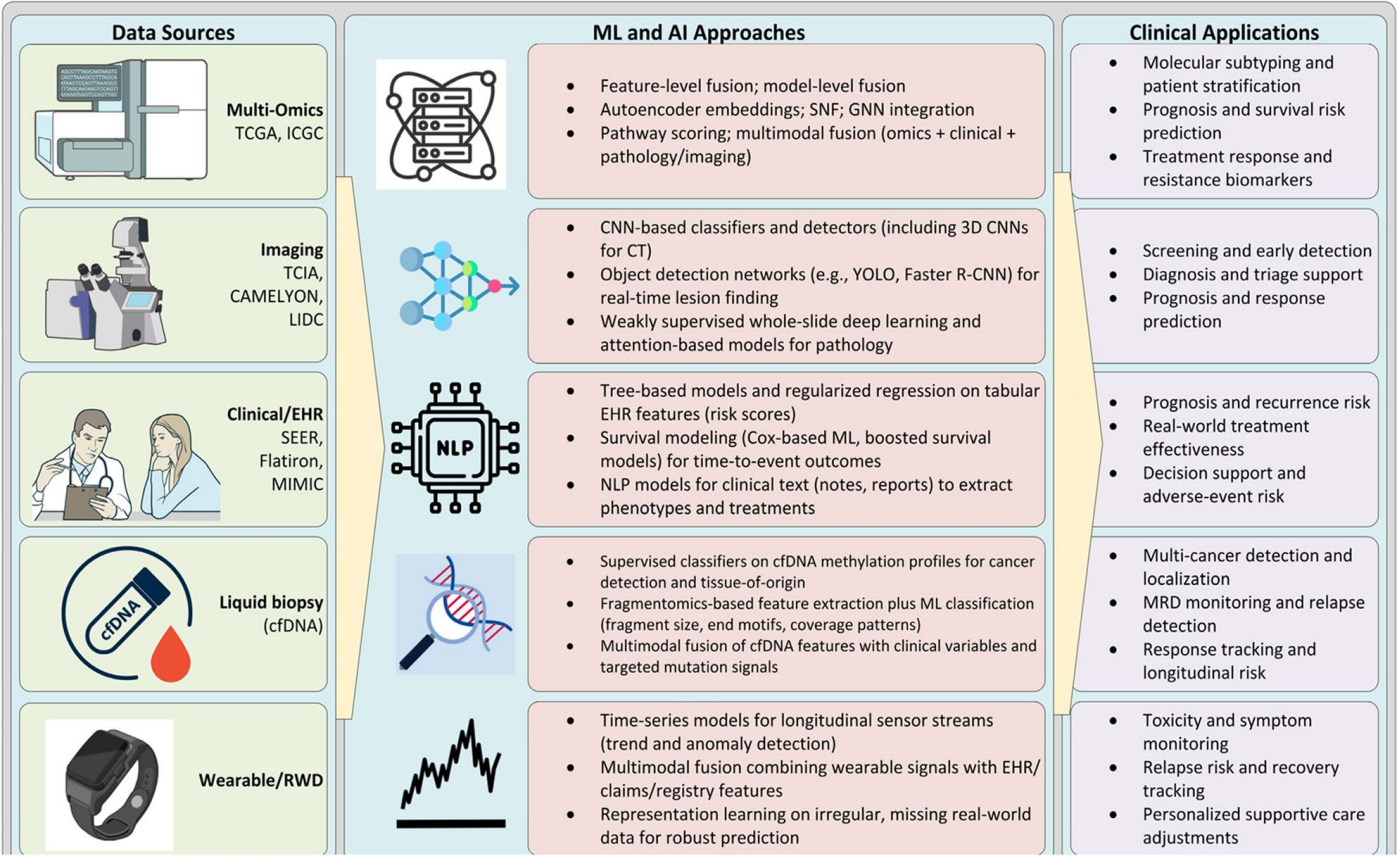
Conceptual framework of ML and AI in cancer research and care. Key data sources (multi-omics, imaging, EHR/clinical data, cfDNA, wearable/real-world data) are linked to common ML/AI approaches and their primary clinical applications, including early detection and diagnosis support, molecular subtyping, prognosis, treatment response, and longitudinal monitoring

The development of an ML and AI in oncology typically follows a structured pipeline designed to ensure data quality, robust modeling, and clinical relevance (Fig. 1). Each stage of the workflow plays a critical role in determining the accuracy, reliability, and translational potential of the final model. AI based multimodal fusion have contributed significantly in these approaches and clinical applications.

An end-to-end ML workflow in oncology typically starts with data acquisition from omics, imaging, and clinical sources, including public repositories such as TCGA or ICGC and institutional cohorts, followed by harmonization across datasets. Data preprocessing then addresses missingness, batch effects, and scaling or encoding of variables, with modality-specific steps such as augmentation for images and standardized variant calling and annotation for genomics. Feature selection or engineering reduces dimensionality and improves interpretability using statistical filters, model-based criteria, or domain knowledge, and may include composite features such as pathway scores. Models are then trained with task-appropriate algorithms and tuned using strategies such as grid, random, or Bayesian search. Generalizability is assessed via cross-validation and independent or external test cohorts using metrics matched to the objective (e.g., AUC, concordance index). Finally, interpretation methods support clinical trust and biological insight, and deployment requires integration into workflows with ongoing monitoring for performance drift and periodic updating as new data and therapies emerge.

As a brief clinical example, a recent multimodal breast cancer survival study used TCGA BRCA data to build an end-to-end prognosis pipeline that mirrors the standard ML workflow. The authors integrated clinical variables with multiple molecular layers (somatic mutations, RNA expression, copy number variation, miRNA) and digitized histopathology images, then compared unimodal models to multimodal deep learning using early and late fusion strategies. After preprocessing and representation learning, models were trained for overall survival prediction and evaluated on held-out data to assess out-of-sample generalization, showing that multimodal integration can improve survival prediction relative to single-modality approaches and providing a practical template for translational risk stratification [29].

### Evaluation metrics

Choosing the right evaluation metric is essential for assessing ML model performance in oncology. Metrics should align with the study objective and account for the unique challenges of biomedical data, such as class imbalance, censoring in survival analysis, and continuous outcome prediction. In classification tasks, accuracy, the proportion of correct predictions, offers a simple summary of performance but can be misleading in imbalanced datasets where one class dominates. To better reflect clinical priorities, precision (positive predictive value) quantifies the proportion of true positives among predicted positives, which matters when false positives lead to unnecessary interventions, while recall (sensitivity) measures the proportion of true positives captured, which is critical in screening where missed cases have severe consequences. The F1-score, the harmonic mean of precision and recall, balances these competing risks when both false positives and false negatives matter. The area under the receiver operating characteristic curve (AUC-ROC) provides an aggregate measure of discrimination across all thresholds and is widely used in binary classification because it is independent of any single decision cutoff [30].

In oncology, the clinical impact of errors is often asymmetric, so evaluation should consider how false negatives and false positives translate into downstream harm and benefit, not discrimination alone. Relying on discrimination alone (e.g., AUC) can mask clinically harmful error trade-offs [31], such as too many missed cancers at the chosen threshold or excessive false alarms that trigger unnecessary tests and treatment. It also ignores calibration, so predicted risks may be systematically over- or under-estimated. Decision curve analysis illustrates this by quantifying net benefit across threshold probabilities in a prostate cancer example, explicitly linking model thresholds to clinical trade-offs [32]. Calibration can be reported with complementary probability-based metrics, including the Brier score [32], which quantifies the mean squared error between predicted probabilities and observed outcomes (lower is better), the Integrated Calibration Index (ICI) with E50 and E90 summaries [33], and binned calibration error measures such as Expected Calibration Error (ECE) and Maximum Calibration Error (MCE) [34].

For time-to-event problems, survival analysis metrics are more appropriate. The concordance index (C-index) assesses agreement between predicted and observed event times; values approaching 1 indicate strong discriminative ability for time-to-event outcomes. Time-dependent AUC extends discrimination assessment across follow-up, capturing how well a model separates high-risk and low-risk individuals at different time horizons, which is important for dynamic risk prediction in oncology.

When outcomes are continuous, regression metrics quantify error magnitude. Mean Absolute Error (MAE) reflects average absolute deviation between predictions and observations, whereas Root Mean Square Error (RMSE) penalizes larger errors more heavily. RMSE is preferable when extreme deviations, for example large tumor size prediction errors, are clinically unacceptable.

### Interpretability and explainable AI

For clinical adoption, interpretability is essential because clinicians must understand why a model supports a given diagnosis, risk estimate, or treatment recommendation. In oncology studies, post hoc explainability tools such as SHAP and LIME, as well as attention or saliency methods in neural networks, are commonly used to audit predictions, identify influential features, and check whether model reasoning aligns with clinical and biological knowledge. Interpretability can also support biological discovery by prioritizing genes, pathways, or microenvironment signals associated with outcomes or therapy response. In a case, a melanoma immunotherapy response model used SHAP to show that tumor immune microenvironment features and mutation-related metrics were key drivers of anti-PD-1 response predictions, helping connect model outputs to actionable biology [35]. Interpretable models not only foster clinician trust but also provide opportunities for biological discovery. For example, an explainable model trained on multi-omics data may highlight novel pathways or genetic interactions associated with treatment resistance. By enabling both clinical utility and scientific insight, explainability has become a cornerstone of machine learning in oncology. In a recent study, it was evaluated an AI test that predicts MSI/MMR status directly from routine H&E colorectal cancer slides, and used tile-level attention heatmaps to highlight the tissue regions driving the model’s predictions, providing a practical interpretability check for pathology-based biomarkers [36].

## Applications in cancer research

Machine learning has had a profound impact across the cancer research pipeline, from early detection to personalized treatment planning. To highlight trends and remaining gaps, Table 1 groups representative studies by higher-level clinical use cases spanning screening and early detection, computational pathology, multi-omics and prognosis, drug response and discovery, and clinical decision support and adaptive therapy (Table 1).

**Table 1:** Representative machine-learning studies in cancer research, annotated by validation setting, clinical maturity, and principal limitation

Clinical domain	Representative study	Task	Validation setting	Clinical maturity	Key finding	Principal limitation
Breast screening	McKinney et al. [37]	Mammography cancer detection	Retrospective external validation	M2	Strong discrimination across held-out test sets	Not a prospective screening implementation
Lung screening	Ardila et al. [38]	LDCT nodule/cancer detection	Retrospective external validation	M2	High sensitivity for lung cancer detection	Retrospective design; workflow impact not tested
Computational pathology	Campanella et al. [39]	Whole-slide tumor detection	Retrospective multi-site validation	M2	High performance on digital pathology tasks	Site and scanner shift remain concerns
Molecular pathology	Bass et al. [36]	AI-based MSI pre-screening in CRC	Multi-centre blinded validation	M2	Strong cross-site diagnostic performance	Not yet an interventional treatment-selection study
Multi-cancer detection	Liu et al. [40]	cfDNA methylation-based detection/localization	Independent validation cohorts	M2	Promising multi-cancer signal detection	Screening impact and population utility still uncertain
Colorectal screening	Repici et al. [41]	Lesion/adenoma detection	Prospective multicenter randomized trial	M4	Increased detection during endoscopy	Surrogate endpoint, not cancer mortality
Prostate cancer diagnosis	Bulten et al. [42]	Targeted prostate biopsy support	Prospective multicenter comparative trial	M2	Improved diagnostic workflow yield	Limited to diagnostic pathway, not long-term outcomes

Clinical maturity categories: M1, retrospective development/internal validation; M2, retrospective external or multicenter validation; M3, prospective observational or silent evaluation; M4, interventional or randomized clinical evaluation

### Early detection and screening

Early cancer detection significantly improves patient survival, as treatment is often most effective at initial stages of disease progression. Machine learning offers tools to identify malignancies earlier and more accurately by recognizing subtle patterns in imaging, genomic, and clinical datasets.

In imaging-based screening, convolutional neural networks trained on mammography have matched or exceeded expert radiologists, with McKinney et al. reporting reduced false positives and false negatives and supporting use as second readers in clinical workflows [37]. For lung cancer, 3D CNNs applied to low-dose CT can improve sensitivity for pulmonary nodules while reducing unnecessary follow-ups, and models that incorporate temporal information across serial scans may help detect subtle changes over time [38]. In colorectal cancer, real-time colonoscopy augmented with object detection networks such as YOLO and Faster R-CNN has increased adenoma detection rates, potentially lowering missed lesions and strengthening prevention strategies [43]. Beyond imaging, liquid biopsy approaches increasingly apply ML to cell-free DNA (cfDNA) signals. ML models trained on cfDNA methylation profiles can distinguish cancer-associated patterns from background and support multi-cancer detection from blood samples [40], while fragmentomics-based approaches that combine cfDNA fragmentation features with sequencing and ML classification have enabled pan-cancer detection and are being evaluated for population-level screening [44].

### Histopathology and computational pathology

With the shift from traditional microscopy to digital histopathology, machine learning now enables automated, large-scale analysis of whole-slide images. Deep learning architectures such as ResNet and Inception have achieved expert-level performance in classifying histopathological images into tumor subtypes, and weakly supervised approaches applied to more than 44,000 slides have shown robust detection of prostate, skin, and breast cancers, substantially reducing pathologists’ workload and improving reproducibility [39]. Beyond classification, models that quantify the tumor microenvironment can measure immune cell infiltration and stromal architecture, capturing spatial relationships that provide clinically relevant insight into prognosis and response to immunotherapy [45]. In addition, ML-derived morphologic features, including nuclear shape and tissue organization, have been linked to survival outcomes, offering cost-effective prognostic biomarkers once digital workflows are in place, which is particularly valuable in resource-limited settings [46].

### Multi-omics integration for subtyping and prognosis

**Fig. 2:**
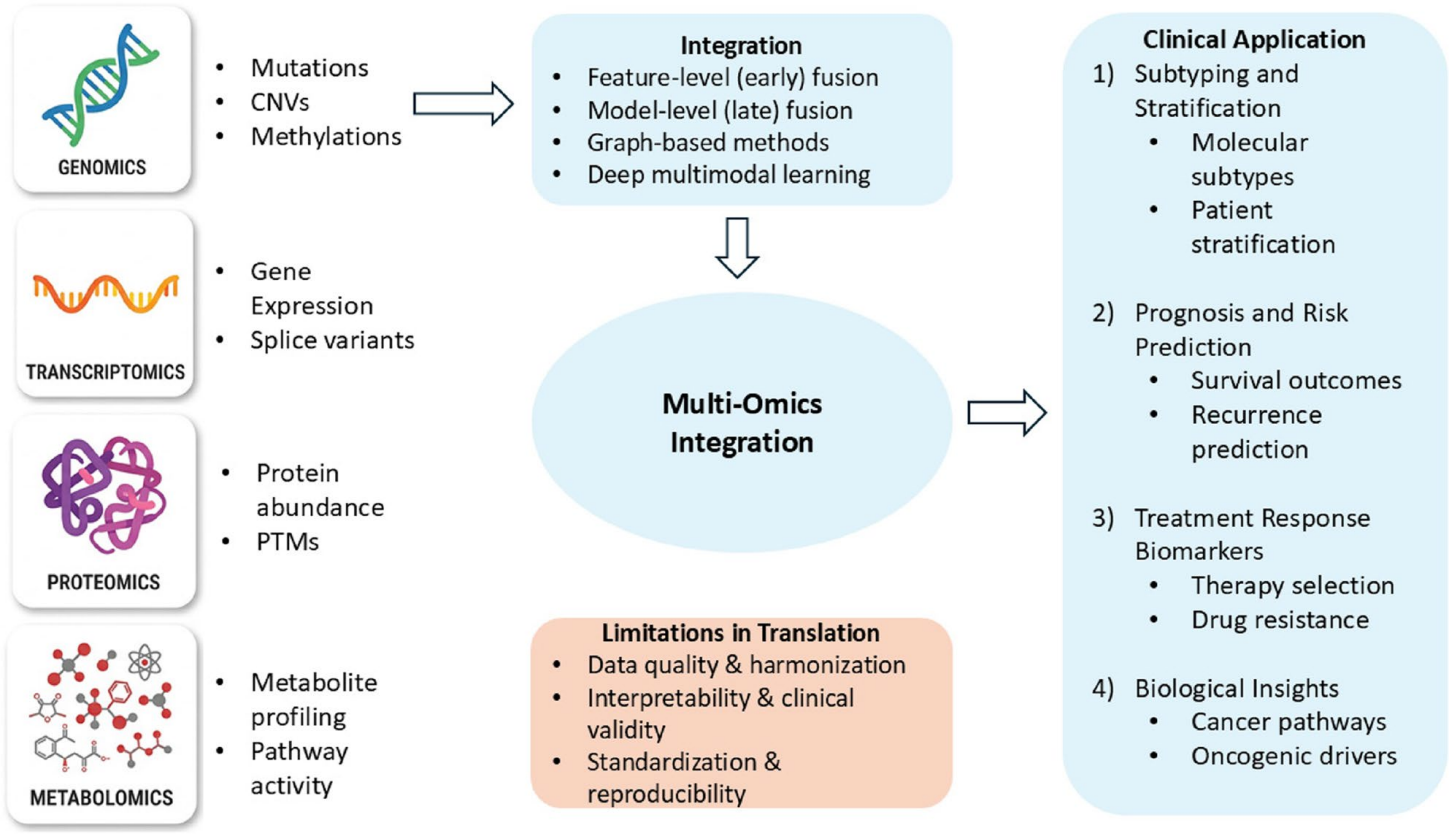
Multi-omics integration in cancer. Molecular layers are integrated using early-fusion, late-fusion, graph-based, and deep multimodal strategies. Most published studies remain retrospective and development-stage, highlighting the need for external validation, prospective testing, and clinically interpretable outputs before routine adoption

Cancer is driven by molecular complexity across genomic, transcriptomic, epigenomic, proteomic, and metabolomic layers, and machine learning provides the computational framework to integrate these heterogeneous data sources for biological insight and clinical utility (Fig. 2). For subtype discovery, TCGA multi-omics integration using methods such as Similarity Network Fusion has identified novel classes with distinct prognostic and therapeutic relevance [47], while autoencoder-based models capture nonlinear dependencies between omics layers, refining subtype calls and enabling detection of rare but clinically meaningful groups [48]. For prognostic modeling, multi-omics ML approaches consistently outperform single-omics analyses in survival prediction, with methods such as random survival forests and deep survival networks proving effective for risk stratification and guiding precision oncology efforts [49].

### Biomarker discovery

Biomarker discovery underpins personalized oncology, and machine learning provides a systematic framework to identify robust signals across molecular and imaging data. On the genomic side, feature selection methods such as LASSO regression and recursive feature elimination have yielded compact yet highly predictive gene panels that outperform broader expression signatures for forecasting recurrence and metastasis [50]. Proteomic signatures discovered with ML have shown promise for earlier detection of ovarian, pancreatic, and lung cancers, creating valuable diagnostic windows, while metabolomic profiles capture tumor-specific metabolic reprogramming that can aid diagnosis and therapy monitoring [51]. In imaging, radiomics transforms MRI and CT scans into high-dimensional quantitative features, and when combined with ML these radiomic biomarkers enable non-invasive prediction of tumor grade, mutation status, and treatment response [52].

### Drug response prediction and pharmacogenomics

Drug response prediction is a cornerstone of precision oncology because it links tumor biology to the therapies most likely to benefit an individual patient. In preclinical settings, large pharmacogenomic resources such as GDSC and CCLE supply training data for models that use genomic features to predict sensitivity to chemotherapies and targeted agents, which helps prioritize candidate drugs before costly laboratory validation [53]. In patient cohorts, integrating tumor molecular profiles with public drug response repositories enables simulation of therapeutic outcomes; deep learning extends this by predicting not only binary response labels but also dose–response curves that support patient-specific regimen selection and dosing decisions [54]. Beyond single agents, reinforcement learning has been used to navigate the enormous search space of combination therapies, identifying synergistic pairs and suggesting schedules that would otherwise take years to uncover experimentally, thereby accelerating the pipeline for novel treatment strategies [55].

### Personalized treatment and clinical decision support

The integration of machine learning into clinical decision-making provides real-time support for oncologists as they tailor treatment strategies to individual patients. For therapy selection, decision support systems such as IBM Watson for Oncology combine natural language processing and machine learning to recommend treatments that align with evidence-based guidelines and current literature, which can reduce variability in care and provide structured recommendations [56]. For adaptive treatment, reinforcement learning models can adjust regimens dynamically, particularly in radiotherapy, by simulating patient responses and modifying doses or schedules so that therapies remain effective as clinical conditions evolve [57]. For risk stratification, machine learning models that combine molecular and clinical variables can categorize patients by recurrence or metastasis risk, helping clinicians decide whether adjuvant therapy is warranted and increasing confidence in personalized treatment plans [58].

## Major data resources and tools for ML in cancer research

The success of machine learning in oncology depends heavily on the availability of large, high-quality datasets and robust computational tools. Below are key resources widely used in the field. These resources can have cohort and measurement biases (site and ancestry skew in TCGA/ICGC and UK Biobank, subtype and treatment-era effects in METABRIC, limited in vivo fidelity in cell lines like GDSC/CCLE, acquisition and annotation variability in imaging datasets such as CAMELYON and LIDC-IDRI/TCIA, and missingness, coding variability, and confounding in real-world EHR registries like SEER, MIMIC-III, and Flatiron), so harmonization and external validation are essential (Tables 2, 3, 4 and 5).

To improve reuse and reproducibility, data and metadata should ideally follow FAIR principles (Findable, Accessible, Interoperable, Reusable), enabling transparent provenance, standardized annotation, and easier cross-cohort integration.

Genomics and multi-omics datasets used in the cancer ML are summarized in Table 2.

**Table 2:** Genomics and multi-omics datasets used in cancer ML

Dataset	Description	Applications	Key translational limitations
The Cancer Genome Atlas (TCGA) [59]	Multi-omics profiles (DNA, RNA, methylation, proteomics) from > 11,000 tumors across 33 cancer types	Subtype discovery, survival prediction, biomarker identification	Retrospective cohort; batch effects, ancestry imbalance, incomplete clinical follow-up, and limited representation of real-world treatment workflows may affect generalizability
International Cancer Genome Consortium (ICGC) [60, 61]	Whole-genome and transcriptome sequencing data from global cancer cohorts	Comparative genomics, rare variant discovery	Cross-cohort heterogeneity, variable sequencing depth, and differences in annotation standards can complicate harmonized ML analyses
METABRIC [62]	Gene expression and clinical data from > 2,000 breast cancer patients	Prognostic modeling, subtype classification	Breast-cancer specific dataset; older assay platforms and treatment-era effects may limit transferability to contemporary clinical practice
Genomics of Drug Sensitivity in Cancer (GDSC) [63]	Drug sensitivity and genomic data for > 1,000 cancer cell lines	Drug response prediction	Cell-line biology may not fully capture tumor microenvironment, immune effects, pharmacokinetics, or patient-level treatment response
Cancer Cell Line Encyclopedia (CCLE) [64]	Genomic, transcriptomic, and pharmacologic profiles of > 1,000 cell lines	Mechanistic studies, therapeutic target discovery	Preclinical cell-line resource; findings require validation in patient-derived models, organoids, xenografts, and clinical cohorts
UK Biobank [65]	Genomic and phenotypic data from 500,000 individuals	Risk prediction, epidemiological studies	Population selection bias, ancestry imbalance, and relatively limited cancer treatment detail may reduce direct applicability to oncology decision support

Key public and large-scale resources (e.g., TCGA, ICGC, METABRIC, GDSC, CCLE, UK Biobank) commonly used for molecular subtyping, survival and risk prediction, biomarker discovery, and drug response modeling. It summarizes widely used genomics and multi-omics resources for cancer subtyping, prognosis, biomarker discovery, and drug response modeling

Imaging datasets used in radiology and computational pathology are summarized in Table 3.

**Table 3:** Imaging datasets used in radiology and computational pathology

Dataset	Description	Applications	Generalizability limitations
CAMELYON16/17 [66]	Annotated histopathology slides for breast cancer metastasis detection	Computational pathology, metastasis detection	Strong benchmark dataset, but narrower than routine pathology case mix; scanner, staining, institution, and slide-preparation differences may affect model performance
LIDC-IDRI [67]	Lung CT scans with nodule annotations	Lung cancer detection and classification	Annotation variability among radiologists and older CT acquisition protocols may limit transferability to current screening programs
TCGA Imaging Archive [68]	MRI, CT, and histology slides linked to genomic data	Multi-modal ML integration	Imaging availability and quality vary across tumor types; linked clinical outcomes may be incomplete, and retrospective collection can introduce selection bias

Representative benchmark datasets (e.g., CAMELYON16/17, LIDC-IDRI, TCGA Imaging Archive) supporting tasks such as metastasis detection, lung nodule classification, and multimodal imaging–omics integration

Representative benchmark datasets (e.g., CAMELYON16/17, LIDC-IDRI, TCGA Imaging Archive) supporting tasks such as metastasis detection, lung nodule classification, and multimodal imaging–omics integration.

Clinical and epidemiological databases for oncology ML are summarized in Table 4.

**Table 4:** Clinical and epidemiological databases for oncology ML

Dataset	Description	Applications	Biases/constraints relevant to deployment
SEER [69]	US cancer incidence, survival, and treatment patterns	Population-level risk modeling	Large population-scale resource, but limited molecular detail, treatment granularity, recurrence information, and real-time clinical workflow data
MIMIC-III [70]	Critical care EHR data	Outcome prediction, comorbidity analysis	ICU-specific population may not generalize to outpatient oncology; missingness, coding variability, and institutional practice patterns can introduce bias
Flatiron Health Database [71]	Real-world oncology EHR data	Treatment effectiveness, survival modeling	Real-world EHR data are valuable but affected by missingness, documentation variability, confounding by indication, and differences in care access

Major population-level and real-world data sources (e.g., SEER, MIMIC-III, Flatiron Health) used for outcome prediction, comorbidity analysis, treatment effectiveness studies, and survival modeling

Computational tools and frameworks commonly used in cancer ML pipelines are summarized in Table 5.

**Table 5:** Computational tools and frameworks commonly used in cancer ML pipelines

Library	Description	Applications	Practical limitations in clinical translation
scikit-learn [72]	Classical machine learning toolkit for regression, classification, clustering, preprocessing, and model selection	Tabular biomedical data, baseline models, feature engineering, cross-validation, benchmarking	Excellent for research and benchmarking, but clinical deployment still requires external validation, monitoring, documentation, and integration with health-system infrastructure
TensorFlow / PyTorch [73, 74]	Deep learning frameworks for building and training neural networks with GPU acceleration and deployment tools	Radiology and histopathology imaging, multi-omics deep learning, NLP of clinical text, transfer learning	Flexible and powerful, but models can be computationally expensive, difficult to interpret, and prone to data leakage or site-specific overfitting if not carefully validated
XGBoost / LightGBM [75, 76]	Gradient boosting decision tree libraries optimized for speed and accuracy; handle missing values and provide feature importance	Risk prediction using EHR and omics, survival modeling variants, feature ranking, imbalanced classification	Strong tabular performance does not guarantee calibration or clinical usefulness; models still require fairness checks, threshold selection, and prospective evaluation
H2O.ai [77]	Automated machine learning platform with hyperparameter tuning, stacking, and scalable distributed training	Rapid prototyping, model comparison across algorithms, production scoring, large-scale pipelines	Automated model selection can obscure methodological choices; careful reporting, interpretability, and independent validation remain necessary
KNIME [78]	Visual workflow analytics platform with drag-and-drop nodes for data wrangling, ML, and integration with R/Python	No-code or low-code reproducible pipelines, team collaboration, deployment of analytic workflows in biomedicine	Useful for workflow reproducibility, but clinical-grade deployment still requires governance, audit trails, regulatory compliance, and performance monitoring

Widely used ML libraries and platforms (scikit-learn, TensorFlow/PyTorch, XGBoost/LightGBM, H2O.ai, KNIME) supporting preprocessing, model development, benchmarking, and scalable deployment in oncology research

## Challenges and limitations in ML for cancer research

Despite the transformative potential of machine learning in oncology, several fundamental and practical challenges hinder widespread clinical translation. Addressing these barriers is essential to ensure safe, equitable, and effective deployment of ML systems in real-world cancer care.

### Data quality and heterogeneity

High-quality, well-curated data are the foundation of successful ML models, yet biomedical datasets are heterogeneous at many levels. Batch effects in omics experiments, introduced by differences in platforms, reagents, or protocols, can obscure true biological signals and confound model training [79]. Even after normalization, residual technical noise can reduce reproducibility across studies. Label noise in clinical datasets, including misdiagnosis, incorrect ICD coding, or inconsistent annotations, degrades model reliability, which is especially problematic for supervised learning where performance depends on label fidelity. Missing data are common in multi-institutional consortia due to variable collection practices or patient attrition, and imputation can introduce bias when missingness is not random. Standardized protocols for acquisition, preprocessing, and harmonization are urgently needed to reduce variability and enable robust cross-study generalization.

### Model interpretability

Deep learning models often achieve state-of-the-art accuracy in diagnostics and prognostics but are frequently perceived by clinicians as black boxes [80]. This opacity reduces trust and slows adoption in high-stakes settings such as cancer diagnosis or therapy selection. Post hoc explanation techniques, including SHAP, LIME, and attention visualizations, can highlight features associated with predictions, but they provide approximations rather than guarantees of the true internal decision process and may be unstable or misleading without careful validation. Improving transparency at design time, for example through intrinsically interpretable architectures, rule-based hybrid models, or sparse networks, may offer more reliable paths to clinical integration. In addition, unclear accountability when an AI-assisted recommendation contributes to harm raises clinical liability concerns and reinforces the need for rigorous governance, documentation, and human oversight.

### Generalizability and bias

Many ML models show limited generalizability due to sampling bias, demographic imbalance, or site-specific training data [81]. Models developed on predominantly Caucasian or urban cohorts may underperform in underrepresented ethnic groups, rural populations, or low-resource settings, raising concerns about health disparities and algorithmic fairness. Site-specific imaging artifacts or idiosyncrasies of electronic health record systems can further encourage overfitting to institutional quirks rather than biologically relevant patterns. Strategies such as domain adaptation, transfer learning, and rigorous cross-site validation are essential to ensure robust performance at deployment.

### Regulatory and ethical considerations

Clinical translation of ML requires navigation of complex regulatory and ethical landscapes. Data privacy laws such as HIPAA in the United States and GDPR in Europe set strict requirements for patient data use [82], motivating technical safeguards including de-identification, federated learning, and differential privacy. ML tools that provide diagnostic or therapeutic recommendations may be regulated as Software as a Medical Device, which entails rigorous evaluation by agencies such as the FDA and EMA. Ethical frameworks must promote transparency, accountability, and non-maleficence, with growing expectations for algorithmic audits, bias assessments, and explainability reports as part of approval and post-market monitoring. Collaboration among clinicians, data scientists, ethicists, and regulators is needed to balance innovation with safety and fairness.

### Computational and resource constraints

Training state-of-the-art ML models, particularly deep architectures, can be computationally expensive and time intensive [83]. Large CNNs and Transformer-based models often require high-performance computing resources with multiple GPUs or TPUs, which may be scarce in academic labs, community hospitals, and low-income regions. Environmental costs linked to energy consumption are an additional concern and have driven interest in energy-efficient AI. Practical solutions include model compression, quantization, distillation, and use of cloud platforms with pay-per-use access to democratize computational resources while controlling costs.

## Future directions in ML for cancer research

The next decade of ML in oncology will likely emphasize improved generalizability and deeper integration into clinical workflows, alongside computational approaches that better capture the complexity of cancer biology and care delivery. As datasets grow in size and diversity, progress will depend not only on technical advances but also on addressing ethical, regulatory, and implementation barriers.

### Federated learning

Federated learning enables collaborative model training across institutions while keeping patient data local [84]. By exchanging model updates rather than raw genomic or clinical records, it can support privacy-preserving learning under frameworks such as HIPAA and GDPR and help capture cancer heterogeneity across sites. Key challenges include non-identical data distributions, variable data quality, and communication efficiency during distributed training.

### Multimodal transformers and patient-level fusion

A key trend is multimodal learning that integrates pathology or radiology representations with clinical variables and molecular layers to form unified patient embeddings rather than simple concatenation or independent models. Recent breast cancer work using TCGA BRCA illustrates this direction by comparing unimodal baselines to multimodal deep learning with early and late fusion across histopathology, clinical variables, and multiple molecular layers for survival prediction [85].

### Real-world and wearable data integration

A growing frontier is integrating real-world data from EHRs, claims, and registries with wearable and sensor streams [86]. These sources can reflect routine clinical practice and enable continuous monitoring of symptoms, activity, and treatment effects, supporting dynamic risk assessment and earlier detection of relapse or toxicity. However, heterogeneity, missingness, and interoperability constraints remain major barriers.

### Graph-based systems biology and therapy discovery

Graph learning is increasingly used to encode biological structure (pathways, interactions) and clinical similarity, enabling models to contextualize alterations within networks rather than treating features independently. GNN-oriented thinking is also spreading into treatment optimization settings where therapy decisions unfold over time, including recent reinforcement learning work that identified personalized intermittent androgen deprivation therapy strategies for prostate cancer [87].

### Explainable AI (XAI) for clinical translation

Interpretability will remain important for safe deployment, but future work is likely to prioritize clinically grounded explanations that are stable, auditable, and aligned with validation requirements, rather than relying solely on post hoc attribution methods [88, 89].

## Discussion

Machine learning has accelerated cancer research by enabling scalable analysis of high-dimensional data and supporting applications ranging from early detection and diagnosis to prognosis and therapy selection. The studies summarized in this review highlight how ML can extract clinically relevant patterns from imaging, multi-omics, and real-world clinical data, reinforcing its growing role in precision oncology.

Translating these advances into routine care requires solving several persistent challenges. First, data quality and heterogeneity remain major barriers to reproducibility and generalizability, particularly across institutions where annotation practices, preprocessing pipelines, and outcome definitions can differ. Standardized data curation, harmonization, and external validation across diverse cohorts are therefore essential to ensure models perform reliably in real-world settings.

Second, interpretability and transparency continue to influence clinical trust, especially for complex deep learning systems. While explainability methods can help contextualize predictions, their outputs must be validated and communicated in clinically meaningful ways alongside uncertainty and failure modes. Third, bias and fairness must be addressed proactively, since models trained on demographically skewed datasets can underperform in underrepresented groups and risk reinforcing disparities. Routine subgroup evaluation, algorithmic auditing, and inclusive dataset development should be treated as core requirements, not optional extras.

Finally, regulatory, ethical, and operational constraints shape whether ML tools can be safely deployed and maintained. Clear governance is needed for monitoring performance drift, handling model updates, and defining accountability in AI-assisted decision making. At the same time, improving access to computational infrastructure and open-source tooling can lower barriers for researchers and promote more transparent, reproducible science.

### Prospective validation and clinical trials

A central challenge in cancer AI is that methodological progress has outpaced high-quality clinical evaluation. Recent meta-research shows that most medical AI evidence remains concentrated in preclinical and retrospective stages. In a 2026 scoping review of 4,667 primary studies, 88.2% were preclinical, and only 2.4% were randomized controlled trials; although neoplasms accounted for 32.5% of all AI studies, oncology represented only 10.6% of the RCTs [90]. This mismatch helps explain why many oncology models report strong discrimination yet still lack the evidence base required for routine implementation.

Post-CONSORT-AI trial evidence also remains limited. A 2024 systematic review of randomized trials for AI interventions identified 65 RCTs overall, with most studies concentrated in gastroenterology and radiology rather than oncology-specific care pathways [91]. The review further showed persistent under-reporting of algorithm version, code or model accessibility, and protocol availability, all of which are critical for reproducibility and clinical trust [91].

In oncology, the most mature examples to date are concentrated in screening and image-guided workflows, whereas many multi-omics, pathology, and treatment-response studies remain retrospective or, at most, externally validated. This suggests that model performance alone should not be equated with clinical readiness. Retrospective internal-validation studies should be interpreted differently from multicenter external validations, prospective silent-rollout studies, and interventional evaluations that test whether AI changes workflow, diagnostic yield, treatment decisions, or patient outcomes [92–94].

This staged progression is consistent with contemporary AI evaluation frameworks. DECIDE-AI provides guidance for early clinical evaluation of AI-based decision-support systems in real-world settings [94], while SPIRIT-AI and CONSORT-AI outline how AI trials should be planned and reported as the field moves toward larger-scale interventional evidence [92, 93]. Future oncology studies should therefore report not only discrimination metrics, but also calibration, failure modes, site-level transportability, human–AI interaction, downstream consequences of false positives and false negatives, and clinically meaningful endpoints such as stage shift, treatment modification, toxicity reduction, or survival benefit [91–94].

## Conclusion

Machine learning is becoming a foundational technology for precision oncology, but clinical impact depends on moving beyond proof-of-concept performance to robust, equitable, and deployable systems. The most important next steps are rigorous external validation across diverse populations, careful management of bias and calibration, clinically meaningful interpretability, and governance frameworks that support safe deployment and monitoring. Take-home message: the models that will matter most in oncology are not only accurate, but also generalizable, transparent, and trustworthy in real-world care.

The next phase of progress in cancer AI will depend less on additional retrospective benchmarks and more on externally validated, prospectively evaluated, and interventional studies that demonstrate reproducibility, safety, workflow benefit, and patient-centered impact.

## Data Availability

Not applicable for this review. No new datasets or code were generated.
